# Superficial Acral Fibromyxoma of the Thumb

**Published:** 2013-01-21

**Authors:** Cindy Wei, Earl J. Fleegler

**Affiliations:** Division of Plastic Surgery, Department of Surgery, University of Medicine and Dentistry, New Jersey—New Jersey Medical School, Newark

**Figure:**
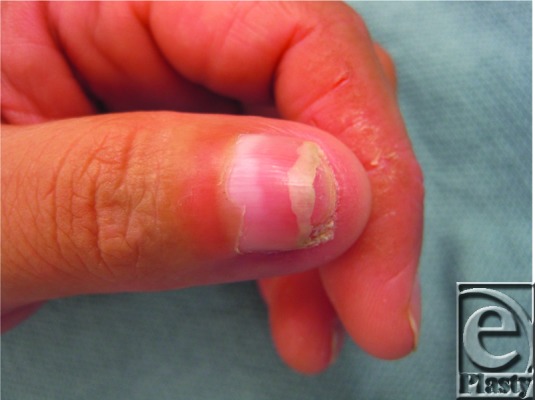


## DESCRIPTION

A 44-year-old man presented with an 8-year history of a slowly enlarging subungual mass of the dominant right thumb.

## QUESTIONS

1. **What are the clinical and histological features of superficial acral fibromyxoma?**
2. **Describe the management of superficial acral fibromyxoma.**
3. **What is the prognosis of superficial acral fibromyxoma?**

## DISCUSSION

Superficial acral fibromyxoma (SAF) is a fibromyxoid tumor with a predilection for the hands and feet, which was first described in 2001.^1^ Since then, numerous case reports and series have been published, suggesting that the tumor may not be as rare as previously thought. Despite this, the entity is not well-recognized by surgeons or pathologists and is not yet described in the World Health Organization blue books of skin or soft tissue tumors.

Superficial acral fibromyxoma most often presents as a slow-growing, nodular mass at acral sites. Previously published series suggest that the toes are more commonly affected than the fingers, with the majority involving the subungual or periungual region; however, the heel, palm, and ankle can also be affected.^1^^-^4 Males are affected approximately twice as often as females, with the average age of presentation in the fifth to sixth decade of life. A history of antecedent trauma is rare. Most tumors are asymptomatic or only mildly tender with pressure, often leading to a delay in patients seeking medical treatment.

Management of these tumors typically involves complete excision to rule out malignancy and prevent recurrence. Although the tumor does not show evidence of aggressive behavior, the presence of cytologic atypia in a small number of cases renders the potential for malignant transformation unclear. Thus far, there have been no reported instances of malignant degeneration in the literature. Periodic follow-up after excision is advised, as the recurrence rate has been reported around 10% to 24%.^2^^,^^4^ This appears more likely with incomplete excision.

The gross appearance of the tumor is typically a firm, nodular, or lobulated mass with a whitish cut surface that can range from solid to gelatinous. The tumor usually involves the dermis, often with extension into the subcutaneous tissue. Occasionally, extension to the fascia or periosteum can cause pressure erosion of the underlying bone. The histologic appearance of SAF consists of fibroblast-like cells with a storiform or fascicular pattern embedded in a predominantly myxoid, myxocollagenous, or collagenous matrix. Increased numbers of ectatic blood vessels and mast cells are commonly seen. Tumor cells typically express CD34, CD99, vimentin, and epithelial membrane antigen and are negative for cytokeratin, muscle, and melanocytic markers.

**Figure 1:**
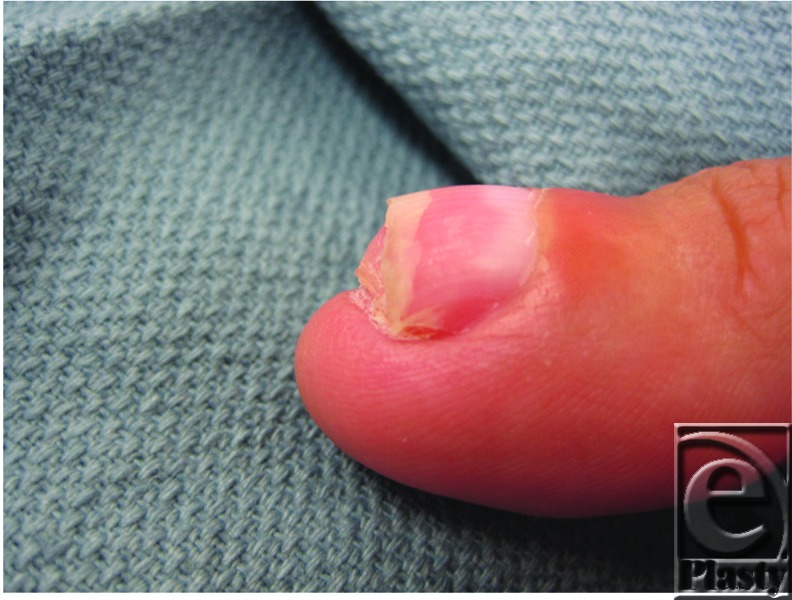
Indolent subungual mass on right thumb.

**Figure 2:**
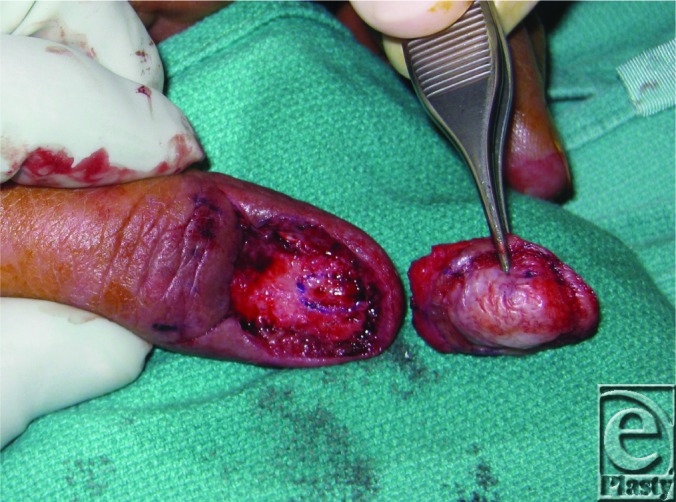
Tumor completely excised. Note suspected area of cortical involvement where cortical osteotomy was performed. Pathology ultimately showed no bony involvement.

**Figure 3:**
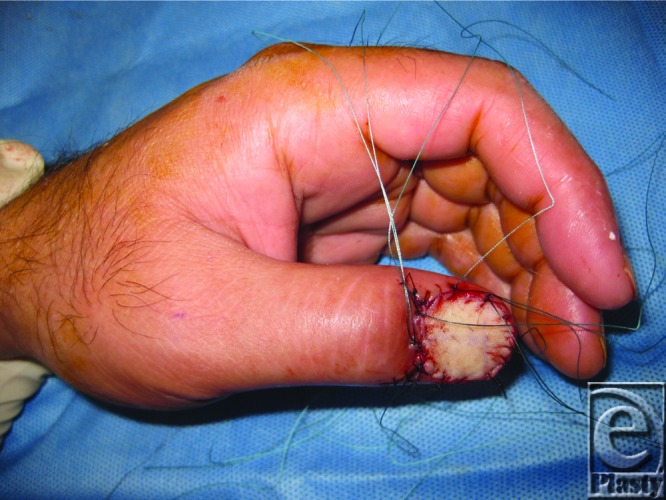
Reconstruction of defect with full-thickness skin graft.

The patient described here presented with an indolent, nontender subungual mass of the thumb with no history of prior trauma (Fig 1). Magnetic resonance imaging revealed a 1.6 × 4.2 × 1 cm^3^ well-defined soft tissue mass overlying the dorsal distal phalanx with apparent infiltration of the dorsal cortex. The distal interphalangeal joint appeared preserved. An incisional biopsy of the mass was performed, which revealed bland fibroblastic cells in a myxoid stroma consistent with SAF. The patient subsequently underwent complete excision of the tumor including the nail matrix (Fig 2). A cortical osteotomy was performed at the distal third of the distal phalanx, where gelatinous material compatible with tumor appeared to extend into a small defect in the underlying cortical bone. Approximately 80% of the periosteum of the distal phalanx was preserved. The defect was reconstructed with a full-thickness skin graft taken from the medial arm (Fig 3). The patient went on to heal uneventfully with no signs of recurrence to this date. Final pathology of the tumor was reported as SAF without involvement of the osteotomized bone. No cytologic atypia or pleomorphism was identified.
